# Association between iron homeostasis and prognosis in patients with chronic obstructive pulmonary disease: a retrospective analysis from MIMIC-IV database

**DOI:** 10.3389/fmed.2025.1610681

**Published:** 2025-06-13

**Authors:** Zhenchao Dong, Chaoqun Xu, Shanjun Yu, Xiaojun Zhang, Jintao Yuan, Liangchen Tang, Li Xie, Jiaqin Zhang, Qi Li, Jian Wang

**Affiliations:** ^1^The People’s Hospital of Danyang and Affiliated Danyang Hospital of Nantong University, Danyang, China; ^2^School of Medicine, Jiangsu University, Zhenjiang, China; ^3^Department of Blood Transfusion, Hainan General Hospital, Hainan Affiliated Hospital of Hainan Medical University, Haikou, China; ^4^Department of Respiratory Medicine, The First Affiliated Hospital of Hainan Medical University, Haikou, China

**Keywords:** chronic obstructive pulmonary disease, long-term mortality, in-hospital mortality, iron homeostasis, MIMIC-IV database

## Abstract

**Introduction:**

Accumulating evidence indicates that inflammatory responses can alter iron-related biomarkers, such as serum iron, ferritin, transferrin, and total iron-binding capacity (TIBC). However, in the context of chronic obstructive pulmonary disease (COPD), characterized by airway inflammation, the relationship between its prognosis and iron-related biomarkers has not been comprehensively assessed.

**Methods:**

Clinical data of 611 COPD patients from the Medical Information Mart for Intensive Care IV (MIMIC-IV) database were retrospectively analyzed. Associations between four iron-related biomarkers—serum iron, ferritin, transferrin, and TIBC—and both long-term and in-hospital mortality in patients with COPD were assessed using the Cox model and the Kaplan-Meier survival analysis. Moreover, receiver operating characteristic curves were used to further evaluate the prognostic predictive ability of these indicators.

**Results:**

The results suggested that higher levels of serum iron and ferritin were significantly associated with poor long-term prognosis in COPD patients. However, higher levels of transferrin and TIBC may reduce the risk of long-term mortality, serving as protective factors. Furthermore, to a certain degree, these four indicators possessed predictive value for both long-term and in-hospital mortality in patients with COPD.

**Conclusion:**

This study underscores the critical connection between iron-related biomarkers and the prognosis of COPD patients, contributing valuable insights for risk stratification and clinical management in this demographic. Future studies, both retrospective and prospective, should investigate the effects of dynamic fluctuations in iron-related biomarkers to enhance the treatment and management of COPD.

## 1 Introduction

Chronic obstructive pulmonary disease (COPD) currently stands as the third leading cause of global mortality, accounting for three million deaths, surpassed only by cardiovascular diseases (1). Many patients with COPD frequently suffer from other significant chronic comorbidities such as heart failure, diabetes, and cancer (2). Despite the plethora of fundamental and clinical studies pertaining to COPD, the extant therapeutic and management modalities for this disease remain constrained.

As an essential trace element, iron is critical for numerous fundamental biological processes, including activities ranging from DNA synthesis to adenosine triphosphate production (3). Low intracellular iron levels impede the performance of proteins that require iron, leading to reduced cellular function and survival (4). However, excessive free iron can catalyze the production of reactive oxygen species (ROS) through the Fenton and Haber-Weiss reactions, thereby damaging tissues and organs (5). External stimuli such as cigarette smoke can disrupt iron homeostasis in lung tissue, and several studies have shown that lung iron regulation is disrupted when COPD occurs (6).

Recent research has revealed a significant prevalence of anemia and systemic iron deficiency among patients with COPD. Two comprehensive cohort studies have demonstrated that an alarming 23–33% of COPD patients suffer from anemia (7, 8). Research also indicates that the prevalence of iron deficiency increases with the severity of COPD (9). Non-anemic iron deficiency is also common in COPD patients, which appears to be caused by inflammation (10).

The perturbation of iron homeostasis with the resulting accumulation of this metal has also been believed to contribute to the pathogenesis of COPD (11). Iron accumulation is frequently observed in the alveolar macrophages of patients with COPD, with the proportion of iron-loaded macrophages rising concomitantly with the progression of disease severity (12). Research has also revealed that compared to healthy individuals, concentrations of iron and associated proteins such as ferritin in the lung tissues and lavage fluids of patients were elevated (13, 14). Increased levels of extracellular ferritin and iron are also significantly associated with COPD exacerbation susceptibility (15). Ferroptosis, a new type of cell death, which is mainly characterized by intracellular iron accumulation and lipid peroxidation, has been considered as a potential therapeutic target for COPD (16). All of these reflect the necessity of monitoring iron homeostasis in COPD.

Regrettably, mortality is a common outcome among patients hospitalized with acute exacerbation of COPD (AECOPD) and severe COPD (17). However, the biomarkers for evaluating the prognosis of these types of COPD are still extremely limited. Acidotic pH, elevated partial pressure of carbon dioxide (PaCO_2_), and reduced arterial oxygen concentration are correlated with increased in-hospital mortality (18). However, the predictive ability of these indicators for the prognosis of AECOPD is still insufficient. Some commonly used scores in the ICU, such as the Acute Physiology and Chronic Health Evaluation (APACHE) II score and pneumonia severity index (PSI), still lack specificity and efficacy for AECOPD and severe COPD (19, 20). Thus, additional biomarkers for prognostic prediction in the acute phase of the disease must be further investigated. Despite being common clinical indicators of iron homeostasis, such as serum iron, ferritin, transferrin, and total iron-binding capacity (TIBC), their relationship with COPD prognosis—particularly in patients with severe COPD—has not been thoroughly evaluated.

In this study, we extracted serum iron, ferritin, transferrin, and TIBC, alongside additional clinical data from patients with COPD recorded in the MIMIC-IV database. Subsequently, we comprehensively evaluated the relationship between these indicators and the prognosis in COPD patients using various statistical methods. Our study thereby supplies a valuable reference for the treatment and management of COPD.

## 2 Materials and methods

### 2.1 Data source

Data for this study were derived from the Medical Information Mart for Intensive Care-IV (MIMIC-IV version 3.0) database, a large dataset of patients admitted to the emergency department or an intensive care unit at the Beth Israel Deaconess Medical Center in Boston, MA. This is an enhanced version of MIMIC-III, which was formerly approved by the institutional review board (21). Chaoqun Xu (ID: 10880658), the co-first author of this study, obtained authorization to extract data from the database following the successful completion of the requisite database usage exam.

### 2.2 Study population

**FIGURE 1:**
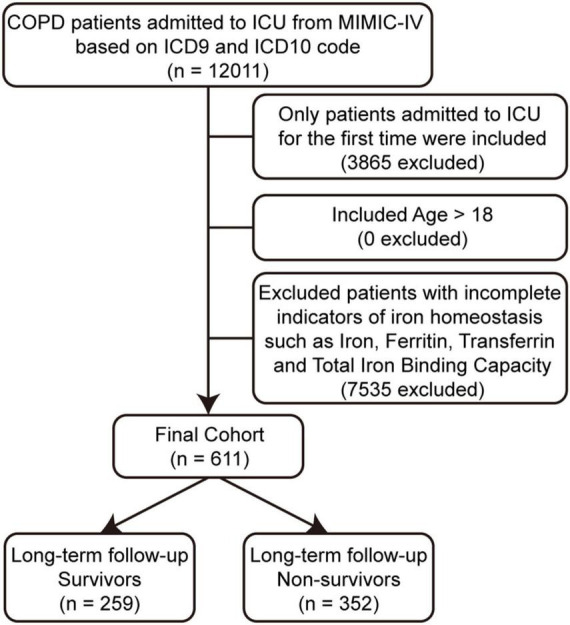
The flow chart of the included population.

This study included patients diagnosed as COPD, based on the ninth and tenth revisions of the International Classification of Diseases (utilizing ICD9 codes: 49120, 49121, 49122, 496 and ICD10 codes: J44, J440, J441, J449). The exclusion criteria were as follows: (1) patients with multiple ICU admissions; (2) patients younger than 18 years; (3) patients lacking complete data on iron homeostasis-related indicators, including serum iron, ferritin, transferrin, and total iron binding capacity (TIBC). Based on the results of long-term follow-up, the patients were divided into survivor and non-survivor groups (Figure 1).

### 2.3 Data extraction and outcomes

Through the use of pgAdmin PostgreSQL tools (version 16.4.0), data on clinical indicators within the first 24 h after admission were obtained, including age, gender, mean blood pressure (MBP), heart rate, respiratory rate, Spo_2_, temperature, ferritin, serum iron, transferrin, TIBC, sequential organ failure assessment (SOFA), and so on. Due to the distinct positive skewness in the distribution of ferritin levels, a log2-transformation was applied to the ferritin data for analytical purposes. Additionally, only indicators with less than 20% missing values were included, and the missing values were imputed using the “mice” package in *R* software. The main outcome in this study was defined as long-term mortality, and the secondary outcome was in-hospital mortality.

### 2.4 Statistical analysis

For continuous variables, those conforming to a normal distribution were depicted as mean ± standard deviation (SD), whereas non-normally distributed variables were represented by median and interquartile range (IQR). The Kolmogorov-Smirnov test was employed to assess the normality of continuous data. Normally distributed continuous variables were analyzed using the *t*-test or analysis of variance while non-normally distributed variables were analyzed using the Mann-Whitney test or the Kruskal-Wallis test. Categorical variables were presented as numbers and percentages, and comparisons were conducted using the chi-square test. The false discovery rate (FDR) correction was used the Benjamini-Hochberg (BH) method. The Kaplan-Meier survival analysis was used to examine the probability of endpoint occurrence in individuals with different levels of iron homeostasis-related indicators, and differences were assessed using the log-rank test. To evaluate the relationship between iron homeostasis-related indicators and both long-term and in-hospital mortality risks, univariate and multivariate Cox proportional hazards regression analyses were conducted. Model 1 did not include any adjustment variables. In Model 2, adjustments were made for age and gender. Model 3 incorporated age, gender, and additional variables that were statistically significant in the univariate Cox regression analysis. Moreover, restricted cubic spline (RCS) was employed to elucidate the non-linear relationships between iron homeostasis-related indicators and both long-term and in-hospital mortality. The receiver operating characteristic (ROC) curves were employed to assess the predictive ability of iron homeostasis-related indicators for in-hospital mortality at 3, 7, and 28 days, as well as long-term mortality at 30, 60, and 90 days. All analyses were conducted using *R* software (version 4.4.1) and SPSS Statistics (version 26.0), with a significance threshold of *P* < 0.05.

## 3 Results

### 3.1 Baseline characteristics

This study included a total of 611 patients with COPD (Table 1). The median age of these patients was 71.3 (IQR, 63.15–80.73) years, and 261 were female, accounting for 42.72% of the total. Based on the results of long-term follow-up, we divided COPD patients into the survivor group and the non-survivor group. The median time of long-term follow-up was 15.8 (IQR, 6.9–87.71) days. The median time of in-hospital follow-up was 8.88 (IQR, 5.63–15.54) days. In addition, patients in the non-survivor group were significantly older than those in the survivor group (*P* < 0.001). Ferritin levels were higher in the non-survivor group than in the survivor group (*P* < 0.05). There was no significant difference in serum iron levels between the two groups (*P* = 0.164). In contrast, the levels of transferrin and TIBC were significantly lower in the non-survivor group compared to the survivor group (*P* < 0.05).

**TABLE 1:** Baseline characteristics of the study population.

Characteristics	Total (*n* = 611)	Survivors (*n* = 259)	Non-survivors (*n* = 352)	*P*-value	FDR
Age, years	71.30 (63.15–80.73)	69.24 (61.66–77.89)	73.64 (64.89–81.92)	<0.001	<0.001
Female, *n* (%)	261 (42.72)	121 (46.72)	140 (39.77)	0.103	0.217
HR, beats/min	84.19 (75.73–96.18)	83.46 (75.83–94.03)	85.06 (75.71–97.24)	0.260	0.309
RR, breaths/min	19.48 (17.09–22.27)	19.19 (17.02–22.16)	19.67 (17.21–22.38)	0.211	0.284
MBP, mmHg	75.64 (70.03–82.57)	77.08 (71.19–84.83)	74.22 (69.51–80.68)	0.001	0.004
Spo_2_,%	96.07 (94.63–97.54)	96.08 (94.57–97.54)	96.02 (94.76–97.50)	0.923	0.933
Temperature, °C	36.75 (36.56–36.97)	36.78 (36.58–36.97)	36.75 (36.55–36.95)	0.047	0.105
Ferritin (log2), ng/mL	7.88 (6.52–9.14)	7.64 (6.27–8.91)	7.97 (6.64–9.27)	0.018	0.045
Serum iron, μg/dL	38.00 (22.00–66.50)	37.00 (21.00–63.00)	39.00 (23.00–71.00)	0.164	0.284
Transferrin, mg/dL	196.00 (149.50–244.00)	200.00 (163.50–252.00)	190.00 (139.50–233.00)	0.003	0.009
TIBC, μg/dL	255.00 (194.50–317.00)	260.00 (212.50–328.00)	247.00 (181.25–303.00)	0.003	0.009
WBC, 10^9^/L	10.50 (7.21–14.45)	10.20 (7.25–13.75)	10.75 (7.22–15.44)	0.168	0.284
Platelets, 10^9^/L	202.67 (136.63–277.63)	211.00 (151.75–277.25)	197.88 (130.90–277.56)	0.202	0.284
Hemoglobin, g/dL	9.08 (7.90–10.60)	9.60 (8.40–11.04)	8.78 (7.65–10.06)	<0.001	<0.001
Neutrophils, 10^9^/L	8.27 (5.63–12.79)	8.12 (5.56–11.83)	8.29 (5.67–13.99)	0.222	0.284
Lymphocytes, 10^9^/L	0.86 (0.47–1.53)	0.96 (0.48–1.62)	0.82 (0.45–1.44)	0.188	0.284
Glucose, mg/dL	132.67 (110.75–170.67)	130.17 (110.88–169.13)	134.50 (110.00–170.72)	0.908	0.933
Calcium, mg/dl	8.50 (7.99–8.90)	8.50 (7.98–8.90)	8.50 (8.00–8.95)	0.636	0.711
Chloride, mEq/L	101.00 (96.50–105.00)	101.67 (97.13–105.00)	101.00 (96.00–105.00)	0.163	0.284
Sodium, mEq/L	138.00 (134.50–140.67)	138.25 (135.33–140.67)	137.88 (134.25–140.67)	0.174	0.284
Potassium, mEq/L	4.30 (3.95–4.80)	4.27 (3.90–4.68)	4.35 (3.99–4.90)	0.017	0.045
Creatinine, mg/dL	1.26 (0.83–2.18)	1.05 (0.73–1.56)	1.44 (0.90–2.54)	<0.001	<0.001
BUN, mg/dL	28.50 (17.63–52.13)	22.50 (15.75–39.17)	36.33 (19.67–60.50)	<0.001	<0.001
AST, IU/L	36.00 (21.00–77.50)	35.50 (21.00–77.00)	37.00 (20.75–77.25)	0.933	0.933
ALT, IU/L	22.00 (13.50–52.33)	21.00 (14.00–57.50)	22.00 (13.38–51.25)	0.815	0.885
PaO_2_, mmHg	75.00 (50.50–104.58)	80.00 (53.50–104.08)	72.00 (49.63–104.83)	0.219	0.284
PaCO_2_, mmHg	44.25 (37.00–51.13)	44.67 (38.00–51.83)	43.88 (36.00–51.00)	0.232	0.284
SOFA, score	5 (3–7)	4 (2–6)	5 (3–8)	<0.001	<0.001
Myocardial infarct, *n* (%)	150 (24.55)	60 (23.17)	90 (25.57)	0.557	0.641
Heart failure, *n* (%)	339 (55.48)	129 (49.81)	210 (59.66)	0.019	0.045
Malignant cancer, *n* (%)	113 (18.49)	22 (8.49)	91 (25.85)	<0.001	<0.001
Renal disease, *n* (%)	216 (35.35)	60 (23.17)	156 (44.32)	<0.001	<0.001
Liver disease, *n* (%)	126 (20.62)	41 (15.83)	85 (24.15)	0.016	0.045
Diabetes, *n* (%)	228 (37.32)	89 (34.36)	139 (39.49)	0.226	0.284
MV, *n* (%)	250 (40.92)	98 (37.84)	152 (43.18)	0.213	0.284
Hospital stays, days	8.88 (5.63–15.54)	8.05 (5.54–13.98)	9.68 (5.67–16.83)	0.141	0.282
In-hospital mortality, *n* (%)	120 (19.64)	0 (0.00)	120 (34.09)	<0.001	<0.001
Long-term follow-up, days	15.80 (6.90–87.71)	8.05 (5.54–13.98)	54.62 (13.18–262.77)	<0.001	<0.001

Data are presented as numbers with percentage or as median with interquartile range. FDR, False discovery rate; HR, Heart rate; RR, Respiratory rate; MBP, Mean blood pressure; SpO_2_, Saturation of peripheral oxygen; TIBC, Total iron binding capacity; WBC, White blood cell; BUN, Blood urea nitrogen; AST, Aspartate aminotransferase; ALT, Alanine aminotransferase; SOFA, Sequential organ failure assessment; MV, Mechanical ventilation.

### 3.2 The results of the Kaplan-Meier survival analysis

**FIGURE 2:**
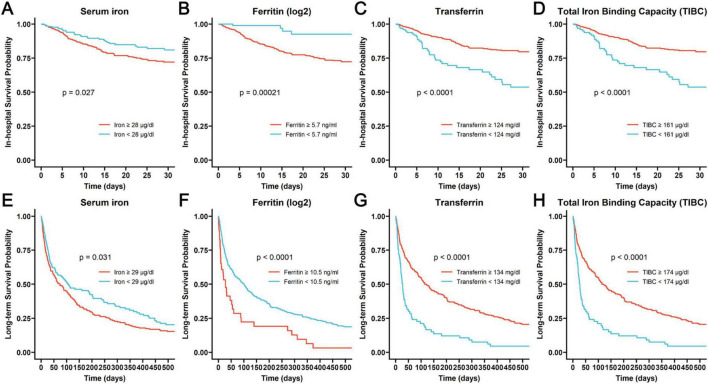
The Kaplan-Meier curves assessing the probability of in-hospital survival associated with different levels of iron **(A)**, ferritin **(B)**, transferrin **(C)** and TIBC **(D)**, and the Kaplan-Meier curves revealing the probability of long-term survival related to different levels of iron **(E)**, ferritin **(F)**, transferrin **(G)**, and TIBC **(H)**.

We employed the “surv_cutpoint” function from the “survminer” package in *R* software to identify the optimal cut-off point for each iron homeostasis-related indicator. Following this, we divided COPD patients into groups with higher and lower values. The Kaplan-Meier survival curves of these groups were then compared using the log-rank test. As shown in Figure 2, higher levels of iron and ferritin as well as lower levels of transferrin and TIBC were associated with both poor in-hospital prognoses and long-term outcomes (log-rank *P* < 0.05).

### 3.3 Univariate and multivariate analyses

**FIGURE 3:**
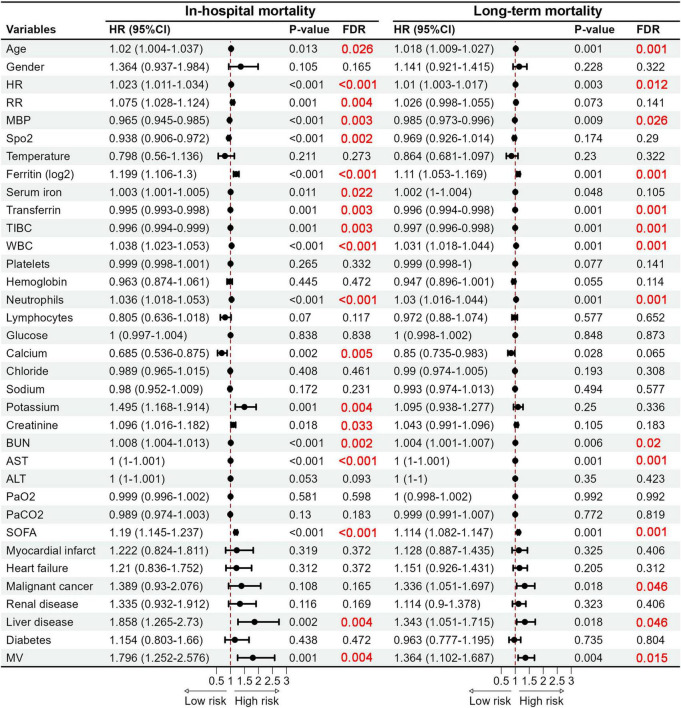
Forest plots of hazard ratios obtained by the univariate Cox regression analysis revealing significant predictors of in-hospital mortality and long-term mortality. HR, Heart rate; RR, Respiratory rate; MBP, Mean blood pressure; SpO_2_, Saturation of peripheral oxygen; TIBC, Total iron binding capacity; WBC, White blood cell; BUN, Blood urea nitrogen; AST, Aspartate aminotransferase; ALT, Alanine aminotransferase; SOFA, Sequential organ failure assessment; MV, Mechanical ventilation.

We further used the Cox Proportional Hazard Model to identify the connection between iron homeostasis-related indicators and the in-hospital mortality as well as long-term mortality in COPD patients. Univariate Cox analysis indicated that heightened serum iron and ferritin levels augmented the risk of in-hospital and long-term mortality. Conversely, significant reductions in the risk of both in-hospital and long-term mortality were linked to elevated transferrin and TIBC levels (Figure 3). Subsequently, we incorporated confounders to execute the multivariate Cox regression analysis. Upon analyzing iron (Table 2), ferritin (Table 3), transferrin (Table 4), and TIBC (Table 5) as continuous variables, in Model 1 and Model 2, all four indicators were significant risk factors for in-hospital and long-term mortality (*P* < 0.05). However, in Model 3, which adjusted all confounders, all four indicators were not important risk factors for in-hospital mortality (*P* > 0.05). Ferritin (HR, 1.072; 95% CI, 1.014–1.133; *P* = 0.015), transferrin (HR, 0.997; 95% CI, 0.995–0.999; *P* = 0.001), and TIBC (HR, 0.998; 95% CI, 0.996–0.999; *P* = 0.001) significantly influenced the risk of long-term mortality, except for iron (*P* > 0.05). After transforming the four indicators into categorical variables based on interquartile ranges, fourth quartile level of serum iron (Table 2) were associated with a higher risk of in-hospital mortality in Model 1 (HR, 1.791; 95% CI, 1.059–3.028; *P* = 0.030) and Model 2 (HR, 1.857; 95% CI, 1.094–3.151; *P* = 0.022). The fourth and third quartile levels of iron were related to a higher risk of long-term mortality in Model 2 (HR, 1.372; 95% CI, 1.018–1.848; *P* = 0.038) and Model 3 (HR, 1.454; 95% CI, 1.053–2.007; *P* = 0.023), respectively. The second and fourth quartile levels of ferritin (Table 3) significantly increased the risk of in-hospital mortality in Model 1 and Model 2. However, only the fourth quartile levels of ferritin significantly increased the risk of long-term mortality in Model 1 (HR, 1.579; 95% CI, 1.167–2.136; *P* = 0.003) and Model 2 (HR, 1.585; 95% CI, 1.162–2.160; *P* = 0.004). The second, third, and fourth quartile levels of transferrin and TIBC were associated with a lower risk of in-hospital mortality in Model 1 and Model 2 (Tables 4, 5). Notably, all quartile levels of transferrin and TIBC decreased the risk of long-term mortality in Model 1, Model 2 and Model 3 (HR < 1; *P* < 0.05). These results indicated that iron homeostasis may play a pivotal role in the long-term prognosis, rather than in the short-term, in-hospital prognosis of COPD patients.

**TABLE 2:** Cox proportional hazard ratios for serum iron.

Parameter	Model 1			Model 2			Model 3		
	HR	95% CI	*P*-value	HR	95% CI	*P*-value	HR	95% CI	*P*-value
In-hospital mortality									
Iron, μg/dL	1.003	1.001–1.005	0.011	1.003	1.001–1.005	0.004	1.001	0.998–1.004	0.492
**Quartile**									
Q1	Ref	Ref	Ref	Ref	Ref	Ref	Ref	Ref	Ref
Q2	1.416	0.820–2.447	0.212	1.383	0.801–2.389	0.245	1.174	0.657–2.097	0.588
Q3	1.518	0.872–2.642	0.140	1.553	0.892–2.705	0.120	1.432	0.797–2.572	0.230
Q4	1.791	1.059–3.028	0.030	1.857	1.094–3.151	0.022	1.496	0.838–2.669	0.173
**Long-term mortality**									
Iron, μg/dL	1.002	1.000–1.004	0.048	1.002	1.000–1.004	0.014	1.001	0.999–1.003	0.294
**Quartile**									
Q1	Ref	Ref	Ref	Ref	Ref	Ref	Ref	Ref	Ref
Q2	1.233	0.914–1.663	0.170	1.225	0.908-1.654	0.184	1.250	0.911–1.715	0.168
Q3	1.247	0.919–1.693	0.157	1.334	0.980-1.816	0.067	1.454	1.053–2.007	0.023
Q4	1.286	0.957–1.729	0.095	1.372	1.018-1.848	0.038	1.382	0.995–1.920	0.053

Patients were divided into four groups according to the quartile range of iron: Q1 ≤ 22 μg/dL, 22 μg/dL < Q2 ≤ 38μg/dL, 38 μg/dL < Q3 ≤ 66.5 μg/dL, Q4 > 66.5 μg/dL. Model 1: unadjusted; Model 2: adjusted for gender and age; Model 3: adjusted for the variables in Model 2 and further for heart rate, respiratory rate, mean blood pressure, Spo_2_, white blood cell, neutrophils, calcium, potassium, creatinine, blood urea nitrogen, aspartate aminotransferase, sequential organ failure assessment, liver disease and mechanical ventilation.

**TABLE 3:** Cox proportional hazard ratios for ferritin (log2).

Parameter	Model 1			Model 2			Model 3		
	HR	95% CI	*P*-value	HR	95% CI	*P*-value	HR	95% CI	*P*-value
**In-hospital mortality**									
Ferritin (log2), ng/mL	1.199	1.106–1.300	<0.001	1.226	1.126–1.334	<0.001	1.055	0.958–1.162	0.273
**Quartile**									
Q1	Ref	Ref	Ref	Ref	Ref	Ref	Ref	Ref	Ref
Q2	1.887	1.036–3.439	0.038	1.840	1.007–3.361	0.047	1.274	0.684–2.375	0.445
Q3	1.698	0.922–3.127	0.089	1.701	0.919–3.151	0.091	1.284	0.675–2.443	0.446
Q4	2.738	1.542–4.862	0.001	2.862	1.596–5.132	<0.001	1.301	0.693–2.445	0.413
**Long-term mortality**									
Ferritin (log2), ng/mL	1.110	1.053–1.169	<0.001	1.116	1.058–1.177	<0.001	1.072	1.014–1.133	0.015
**Quartile**									
Q1	Ref	Ref	Ref	Ref	Ref	Ref	Ref	Ref	Ref
Q2	1.319	0.973–1.790	0.075	1.282	0.942–1.744	0.114	1.048	0.753–1.459	0.780
Q3	1.201	0.881–1.638	0.247	1.215	0.887–1.664	0.225	1.125	0.808–1.567	0.485
Q4	1.579	1.167–2.136	0.003	1.585	1.162–2.160	0.004	1.304	0.934–1.821	0.119

Patients were divided into four groups according to the quartile range of ferritin (log2): Q1 ≤ 6.52 ng/mL, 6.52 ng/mL < Q2 ≤ 7.88 ng/mL, 7.88 ng/mL < Q3 ≤ 9.14 ng/mL, Q4 > 9.14 ng/mL. Model 1: unadjusted; Model 2: adjusted for gender and age; Model 3: adjusted for the variables in Model 2 and further for heart rate, respiratory rate, mean blood pressure, Spo_2_, white blood cell, neutrophils, calcium, potassium, creatinine, blood urea nitrogen, aspartate aminotransferase, sequential organ failure assessment, liver disease and mechanical ventilation.

**TABLE 4:** Cox proportional hazard ratios for transferrin.

Parameter	Model 1			Model 2			Model 3		
	HR	95% CI	*P*-value	HR	95% CI	*P*-value	HR	95% CI	*P*-value
**In-hospital mortality**									
Transferrin, mg/dL	0.995	0.993–0.998	0.001	0.995	0.992–0.998	0.001	0.999	0.996–1.002	0.357
**Quartile**									
Q1	Ref	Ref	Ref	Ref	Ref	Ref	Ref	Ref	Ref
Q2	0.461	0.280–0.758	0.002	0.417	0.252–0.689	0.001	0.686	0.395–1.190	0.180
Q3	0.498	0.307–0.808	0.005	0.442	0.270–0.725	0.001	0.770	0.439–1.348	0.360
Q4	0.540	0.331–0.881	0.014	0.535	0.325–0.878	0.013	0.908	0.524–1.571	0.729
**Long-term mortality**									
Transferrin, mg/dL	0.996	0.994–0.998	<0.001	0.996	0.994–0.998	<0.001	0.997	0.995–0.999	0.001
**Quartile**									
Q1	Ref	Ref	Ref	Ref	Ref	Ref	Ref	Ref	Ref
Q2	0.592	0.443–0.792	<0.001	0.537	0.400–0.721	<0.001	0.608	0.445–0.831	0.002
Q3	0.570	0.428–0.759	<0.001	0.528	0.393–0.709	<0.001	0.624	0.450–0.865	0.005
Q4	0.566	0.418–0.768	<0.001	0.547	0.400–0.747	<0.001	0.625	0.442–0.883	0.008

Patients were divided into four groups according to the quartile range of transferrin: Q1 ≤ 150 mg/dL, 150 mg/dL < Q2 ≤ 196 mg/dL, 196 mg/dL < Q3 ≤ 244 mg/dL, Q4 > 244 mg/dL. Model 1: unadjusted; Model 2: adjusted for gender and age; Model 3: adjusted for the variables in Model 2 and further for heart rate, respiratory rate, mean blood pressure, Spo_2_, white blood cell, neutrophils, calcium, potassium, creatinine, blood urea nitrogen, aspartate aminotransferase, sequential organ failure assessment, liver disease and mechanical ventilation.

**TABLE 5:** Cox proportional hazard ratios for total iron binding capacity (TIBC).

Parameter	Model 1			Model 2			Model 3		
	HR	95% CI	*P*-value	HR	95% CI	*P*-value	HR	95% CI	*P*-value
**In-hospital mortality**									
TIBC, μg/dL	0.996	0.994–0.999	0.001	0.996	0.994–0.998	0.001	0.999	0.997–1.001	0.358
**Quartile**									
Q1	Ref	Ref	Ref	Ref	Ref	Ref	Ref	Ref	Ref
Q2	0.461	0.280–0.758	0.002	0.417	0.252–0.689	0.001	0.686	0.395–1.190	0.180
Q3	0.498	0.307–0.808	0.005	0.442	0.270–0.725	0.001	0.770	0.439–1.348	0.360
Q4	0.540	0.331–0.881	0.014	0.535	0.325–0.878	0.013	0.908	0.524–1.571	0.729
**Long-term mortality**									
TIBC, μg/dL	0.997	0.996–0.998	<0.001	0.997	0.995–0.998	<0.001	0.998	0.996–0.999	0.001
**Quartile**									
Q1	Ref	Ref	Ref	Ref	Ref	Ref	Ref	Ref	Ref
Q2	0.592	0.443–0.792	<0.001	0.537	0.400–0.721	<0.001	0.608	0.445–0.831	0.002
Q3	0.570	0.428–0.759	<0.001	0.528	0.393–0.709	<0.001	0.624	0.450–0.865	0.005
Q4	0.566	0.418–0.768	<0.001	0.547	0.400–0.747	<0.001	0.625	0.442–0.883	0.008

Patients were divided into four groups according to the quartile range of total iron binding capacity (TIBC): Q1 ≤ 194 μg/dL, 194 μg/dL < Q2 ≤ 255 μg/dL, 255 μg/dL < Q3 ≤ 317 μg/dL, Q4 > 317 μg/dL. Model 1: unadjusted; Model 2: adjusted for gender and age; Model 3: adjusted for the variables in Model 2 and further for heart rate, respiratory rate, mean blood pressure, Spo_2_, white blood cell, neutrophils, calcium, potassium, creatinine, blood urea nitrogen, aspartate aminotransferase, sequential organ failure assessment, liver disease and mechanical ventilation.

### 3.4 The results of RCS analysis

**FIGURE 4:**
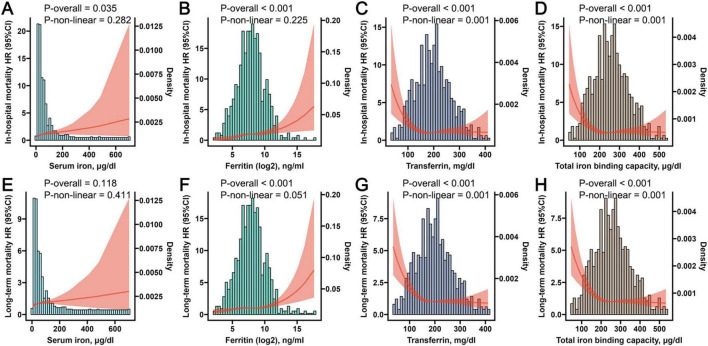
The Restricted Cubic Spline (RCS) plots displaying non-linear correlations between iron **(A)**, ferritin **(B)**, transferrin **(C)**, and total iron binding capacity **(D)** with in-hospital mortality, while simultaneously illustrating the non-linear associations between iron **(E)**, ferritin **(F)**, transferrin **(G)**, and total iron binding capacity **(H)** with long-term mortality.

To ascertain the existence of a non-linear association between the four iron homeostasis-related indicators and both in-hospital and long-term mortality, we employed the RCS analysis. A discernible linear relationship was established between iron, ferritin and the risk of in-hospital mortality, as well as long-term mortality (Figures 4A,B,E,F). However, a non-linear association was observed between transferrin, TIBC, in-hospital mortality, and long-term mortality (Figures 4C,D,G,H). Interestingly, upon reaching certain thresholds, the increase in transferrin and TIBC levels no longer significantly reduced the risks of in-hospital and long-term mortality.

### 3.5 The results of ROC analysis

**FIGURE 5:**
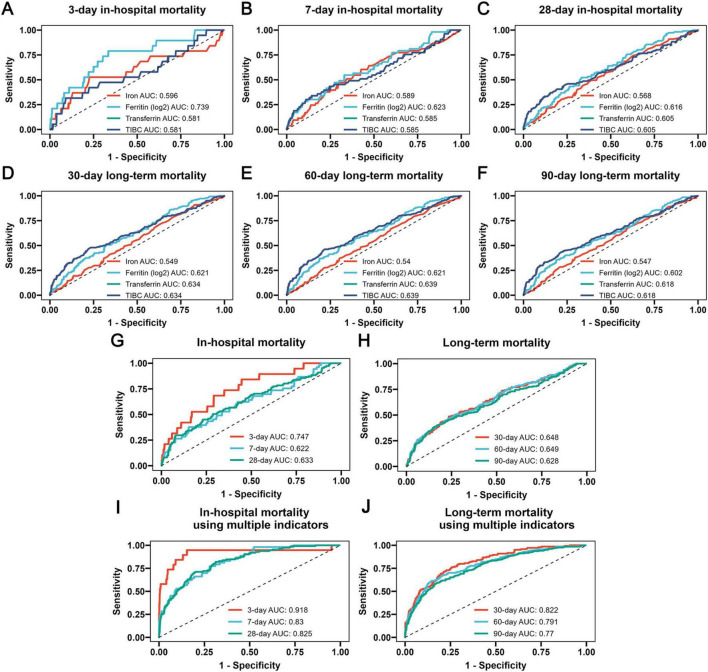
The receiver operating characteristic (ROC) curves revealing the ability of iron homeostasis-related indicators to predict 3-day **(A)**, 7-day **(B)** and 28-day **(C)** in-hospital mortality, as well as 30-day **(D)**, 60-day **(E)** and 90-day **(F)** long-term mortality, the ROC curves revealing the ability of the logistic regression model developed using three iron homeostasis-related indicators such as serum iron, ferritin and transferrin to predict in-hospital **(G)** and long-term **(H)** mortality, and the additional ROC curves revealing the ability of the logistic regression model developed using multiple indicators such as serum iron, ferritin, transferrin, age, heart rate, respiratory rate, MBP, SpO2, WBC, neutrophils, calcium, potassium, creatinine, BUN, AST, SOFA and mechanical ventilation to predict in-hospital **(I)** and long-term **(J)** mortality.

The ROC analysis was utilized to assess the precise predictive value of iron homeostasis-related indicators regarding the prognosis of COPD. Since TIBC can indirectly reflect the level of transferrin, TIBC and transferrin had the same prognostic predictive value for COPD. As shown in Figures 5A–C, the four indicators had certain predictive value for 3-, 7-, and 28-days in-hospital mortality, with ferritin exhibiting the highest predictive value among them (Ferritin 3-day AUC: 0.739; 7-day AUC: 0.623; 28-day AUC: 0.616). The four indicators also had certain predictive value for 30-, 60-, and 90-days long-term mortality (Figures 5D–F), while the AUC values of transferrin and TIBC were the highest (30-day AUC: 0.634; 60-day AUC: 0.639; 90-day AUC: 0.618). Moreover, the combined predictive capability of these four indicators for the prognosis of COPD patients was also assessed. Based on previous analyses indicating that TIBC and transferrin exhibit similar effects, we excluded TIBC from the study and utilized three iron homeostasis indicators to construct a logistic regression model. The ROC results of the model demonstrated favorable predictive value for both in-hospital mortality and long-term mortality (Figures 5G,H). Notably, it showed the highest predictive ability for determining 3-day in-hospital mortality (AUC: 0.747) and 60-day long-term mortality (AUC: 0.649). When iron, ferritin, and transferrin were combined with additional indicators such as age, heart rate, respiratory rate, MBP, SpO_2_, WBC, neutrophils, calcium, potassium, creatinine, BUN, AST, SOFA and mechanical ventilation, the predictive capability of the logistic regression model is further improved (Figures 5I,J).

## 4 Discussion

In this study, we evaluated the association between iron homeostasis-related biomarkers (serum iron, ferritin, transferrin, and TIBC) and long-term and in-hospital mortality in COPD patients from the MIMIC-IV database. Higher levels of iron and ferritin and lower levels of transferrin and TIBC were associated with poor long-term and in-hospital outcomes in COPD patients. However, COX models that adjusted for confounders indicated a tighter correlation between these four indicators and the long-term prognosis. Moreover, the amalgamation of these four indicators demonstrated a degree of predictive value for both short-term in-hospital mortality and long-term mortality.

COPD is one of the diseases that cause a significant global socioeconomic burden. The typical characteristics of COPD are persistent respiratory symptoms and restricted airflow, resulting primarily from small airway disease and alveolar destruction (22). Smoking is widely recognized as one of the risk factors for COPD. Cigarette smoke-induced ROS formation often triggers chronic systemic inflammation, which is considered to be one of the important pathogenesis of COPD (23). Fe3 + can form complexes with the particulate matter deposited from cigarette smoke, accumulating on the surface of the lungs of smokers (24). Studies have shown increased serum ferritin (a metal storage protein) and iron concentrations in smokers, reflecting disruption of iron homeostasis and its accumulation (25). Significant indications robustly suggest an association between COPD and iron homeostasis. Our current study further suggests the importance of iron homeostasis in the long-term prognosis of patients with COPD. The baseline characteristics in this study implied that, among COPD patients who exhibit unfavorable long-term prognoses, there was an increase in serum ferritin levels, coupled with a decrease in both transferrin and TIBC levels. The Kaplan-Meier survival analysis suggested that higher levels of iron and ferritin, along with lower levels of transferrin and TIBC were associated with poor in-hospital and long-term outcomes.

Iron deficiency has also been observed in COPD patients in previous studies (26). Unexpectedly, we did not find that COPD patients with low serum iron and ferritin levels were related to an increased risk of mortality in this study. The rationale behind this phenomenon could pertain to the inability of plasma ferritin and serum iron levels to sufficiently illustrate the cellular iron homeostasis, due to their susceptibility to numerous factors. More research is needed to discover new biomarkers that correctly reflect cellular iron deficiency in COPD patients. Studies have shown that elevated levels of ferritin and serum iron often indicate chronic inflammation (27–29). Moreover, an overabundance of iron can result in lung damage via mechanisms related to ferroptosis (16). Since the subjects of this study were mainly COPD patients admitted to the ICU, these results also suggested that we should pay more attention to elevated iron levels in patients with severe COPD.

Since the patients included in this study exhibited acute exacerbations and severe COPD, acute inflammation significantly impacts iron homeostasis-related indicators. Acute inflammation leads to the destruction of erythrocytes and hepatic cells, resulting in disrupted iron metabolism and the accumulation of iron (30). Elevated ferritin levels are often observed in acute inflammatory conditions (31). Ferritin is frequently regarded as a pro-inflammatory cytokine (32). However, during the acute inflammatory period, transferrin can be inhibited by pro-inflammatory cytokines such as tumor necrosis factor-α (30). These findings align with our conclusion that elevated levels of serum iron and ferritin correlate with a poor prognosis in patients with COPD, whereas higher levels of transferrin and TIBC may lower the risk of mortality.

In this study, both Cox regression analysis and RCS analysis showed that high levels of transferrin and TIBC were associated with a reduced risk of in-hospital and long-term mortality in COPD patients. Previous studies have also shown that COPD individuals have lower levels of transferrin than healthy individuals (33, 34). Recent studies suggest that a decrease in transferrin levels may be related to the worsening of COPD (35). Furthermore, research shows that among all COPD patients, serum transferrin is significantly positively correlated with forced expiratory volume in 1 s (FEV1) (36). These findings suggest that the supplementation of transferrin could potentially improve pulmonary dysfunction in COPD patients. Additionally, the analysis of the ROC curve demonstrated that the model combined by four indicators related to iron homeostasis possessed a robust predictive capacity for both in-hospital and long-term mortality. Overall, this study highlights the importance of detecting iron homeostasis for the daily management of COPD.

This study had several limitations. Firstly, this study concerned patients with COPD admitted to the ICU. Further research is required to ascertain the prognostic value of iron homeostasis in those with stable COPD. Secondly, due to the excessive number of missing values, biomarkers with documented prognostic impact, such as FEV1, C-reactive protein and cardiac troponin, were not included. Thirdly, this single-center clinical retrospective study has potential limitations due to missing data, which could lead to biased results. Future multicenter prospective studies are necessary to further validate these findings. Moreover, this research was limited to examining only the initial levels of iron homeostasis, without exploring the dynamic changes of iron homeostasis. Therefore, further clinical studies are required to understand the relationship between the dynamic changes of these indicators and the prognosis in COPD patients.

## 5 Conclusion

This study extends the applicability of iron homeostasis-related indicators to COPD patients, emphasizing their potential utility as long-term mortality risk stratification indicators especially for severe patients in this demographic. The detection of iron homeostasis is beneficial to the treatment and management of COPD. In the future, new interventions targeting iron homeostasis are needed to improve the prognosis of COPD patients.

## Data Availability

The original contributions presented in the study are included in the article/supplementary material, further inquiries can be directed to the corresponding authors.
